# Alien hand syndrome in AIDS: Neuropsychological features and physiopathological considerations based on a case report

**DOI:** 10.1590/S1980-57642008DN10400016

**Published:** 2007

**Authors:** Leonardo Caixeta, Patrícia Maciel, Juliana Nunes, Larissa Nazareno, Letícia Araújo, Jules Rimet Borges

**Affiliations:** 1 Adjunct Professor. Behavioral Neurology Unit, Hospital das Clínicas, Federal University of Goiás, Brazil.; 2 collaborating physician. Behavioral Neurology Unit, Hospital das Clínicas, Federal University of Goiás, Brazil.

**Keywords:** alien hand syndrome, pathophysiology, toxoplasmosis, AIDS, frontal, parietal, corpus callosum, síndrome da mão alienígena, neuropsicologia, fisiopatologia, neurotoxoplasmose, AIDS, frontal, parietal, corpo caloso

## Abstract

Alien hand syndrome consists of an autonomous motor activity perceived as an involuntary yet purposeful movement, with a feeling of foreignness of the involved limb, commonly associated with a failure to recognize ownership of the limb in the absence of visual cues. A 41 year old left-handed woman, HIV positive, evolved with loss of control in the left hand. Her left hand presented extravolitional movements, as if having a will of its own, not responding to commands such as opening a door or holding an umbrella, but instead groping unneeded objects. She had talked to her hand and even fought it. In addition, other clinical presentations including recent memory loss, hemineglect and dysphoria were observed. Computed tomography revealed a hypodensity area in the right frontal-parietal region, with midline deviation. Considering clinical and epidemiological data, the diagnosis of Central Nervous System (CNS) toxoplasmosis was reached. No previous reports showing association among AIDS, toxoplasmosis and alien hand syndrome were found.

## Abstract (pt)

A síndrome da mãe alienígena caracteriza-se pela presença de atividade motora autônoma involuntária e aparentemente proposital de um membro, acompanhada de uma sensação de estranheza em relação ao mesmo, podendo associar-se a uma dificuldade em reconhecê-lo na ausência de pistas visuais. Uma paciente de 41 anos, HIV positiva, com dominância manual sinistra, evoluiu com perda do controle sobre a mão esquerda, que passou a ter “vontade própria”, não obedecendo ao comando de, por exemplo, abrir uma porta ou pegar uma sombrinha, mas agarrando objetos, inúteis para a paciente no momento, que ela não conseguia soltar facilmente. Além disso, a paciente referia outras alterações, como perda de memória recente, heminegligência e disforia. A tomografia computadorizada mostrou área de hipodensidade em região fronto-parietal direita, com desvio de linha média. Considerando dados clínicos e epidemiológicos, foi estabelecido diagnóstico de neurotoxoplasmose. Não se encontram relatos da associação entre AIDS, neurotoxoplasmose, síndrome da mãe alienígena.

The phenomenon of the alien hand has been known since 1908, when Kurt Goldstein^1,2^ described the case of a 57-year-old woman who suffered a stroke and there after perceived her left hand as having a will of its own. Only in 1972, did Brion and Jedynak^3^ propose the term *alien hand* (*la main étrangère*) to describe this type of clinical presentation in patients with midline brain tumors who exhibited a denial of ownership of one of their hands. The main feature of all these cases was the individual’s perception of the affected hand as being out of volitional control while performing simple to complex extravolitional motor activities.^4^

This syndrome’s etiology^5^ was primarily linked to callosal tumors^3^, but also surgical callosotomy^4^, infarction of the medial frontal cortex, occipitotemporal lobe and thalamus^7,10^, infection^8^, and corticobasal degeneration^7,9^.

Feinberg et al.^11^ proposed that the alien hand syndrome has two main subtypes: callosal and frontal. This feature is easy to explain since these areas are clearly closely related to motor planning and its final pathways.^1^

The callosal form includes complex willed motor acts by the *nondominant* hand, where patients rarely present a grasp reflex or compulsive tool manipulation but frequently exhibit inter-manual conflict, in which one hand acts at cross-purposes with the other.^6^ The frontal type affects the *dominant* hand and includes grasp reflex, impulsive groping toward objects or, in Denny-Brown’s terminology,^12^ *magnetic apraxia*, in which the affected hand reaches toward and grasps objects as if drawn to them by a magnet with subsequent release of the objects proving difficult, as well as compulsive tool manipulation. As the medial frontal lobe damage often is associated to damage to the corpus callosum, frontal type cases may also present callosal form signs. Cases of damage restricted to the callosum however, tend not to show frontal alien-hand signs.^20^

Several cases of alien hand syndrome have been reported after posterior lesions resulting either from corticobasal degeneration involving primarily posterior cortical degeneration or from cerebrovascular disease.^9,13,16-19^ Generally, though not exclusively, these cases have involved the non-dominant limb. The sustained involuntary movements are typically non-purposeful and non-conflictual, and include such behaviors as arm levitation and finger writhing.^9,19^

We report the first case of alien hand syndrome in a patient with central nervous system toxoplasmosis, using it as a basis for discussing some neuropsychological features and physiopathological mechanisms related to this syndrome. The patient gave informed consent to report her case.

## Case report

A 41 year-old, left-handed, HIV positive, white woman was admitted at our university hospital in May 2005, having presented right hemicranial headache for one month, associated with fever, shivers, vomiting and asthenia. She also reported a pulling sensation involving her mouth on the left side with local erythema and drooling. During the same period, she developed brief episodes of left hand weakness associated with dysarthria. Two weeks later, the patient lost control of her left hand, mainly at night where upon waking her left hand was grasping her right arm.

Subsequently, her dominant hand presented extravolitional movements, as if having a will of its own, not responding to volitional commands such as opening a door or holding an umbrella, but groping unneeded objects and having difficulty releasing them. The patient often found her left hand closed, in front of her face, as if it were “looking” at her. She was afraid of her hand: “it seemed to be a monster”. It also scratched and hurt the patient’s face and body. She had talked to her hand and even fought it. Other clinical presentations including recent memory loss, hemineglect (patient deployed only half of visual field) and dysphoria in the context of depressed organic mood were also verified. On physical evaluation, the patient had central hemifacial palsy. These symptoms lasted for two weeks and had a significant impact on the patient’s life, disappearing only after etiological treatment of toxoplasmosis.

**Figure 1:**
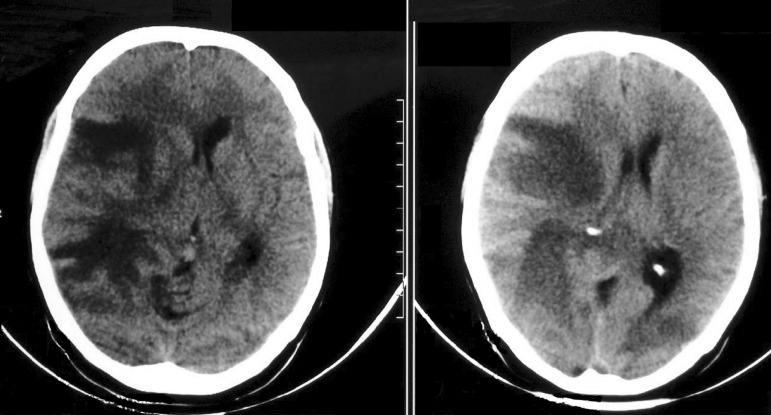
Head computed tomography showing a hypodensity area in the right frontal-parietal region with mass effect and midline deviation.

Hemogram showed leucopenia, with lymphocytopenia. Cerebrospinal fluid evaluation showed slight cell increase, preponderance of mononuclear cells, elevated protein level, without glucose consumption. Serologies for cryptococcosis, syphilis, neurocysticercosis and cytomegalovirus were negative. Cranial computed tomography revealed a hypodense area in the right frontal-parietal region with midline deviation (Figure 1).

Considering clinical, laboratorial and epidemiological data, CNS toxoplasmosis was the most likely diagnosis. The patient was empirically treated with sulfadiazine, pyrimethamine, hidantoine, amitriptyline, leucovorin, mannitol and dexamethasone. This treatment was successful, confirming the hypothesis of Toxoplasmosis. The patient evolved with progressive improvement of symptoms, including alien-hand signs discharged from hospital.

## Discussion

Alien hand syndrome is one of the most intriguing neurological syndromes. It is defined as unwilled, uncontrollable, but seemingly purposeful movements of an upper limb. Two major criteria for the diagnosis are complaint of limb and complex, autonomous, involuntary motor activity, not part of an identifiable movement disorder. A verbally expressed feeling that the movements are not under self control and personification of the arm also occur.^6,13^ According to this definition, our patient fulfilled the criteria for alien hand syndrome. Personification of the arm was a marked feature in this case, since the patient became frightened of her “threatening arm”.

The signs she presented were mainly related to the frontal form of alien hand syndrome (dominant hand was affected, grasp reflex and impulsive groping were seen), but features of callosal or even posterior alien hand syndrome could not be ruled out, being consistent with the CT image which showed a lesion with extensive edema affecting frontoparietal regions and probably callosal fibers, since there was midline deviation.

**Figure 2:**
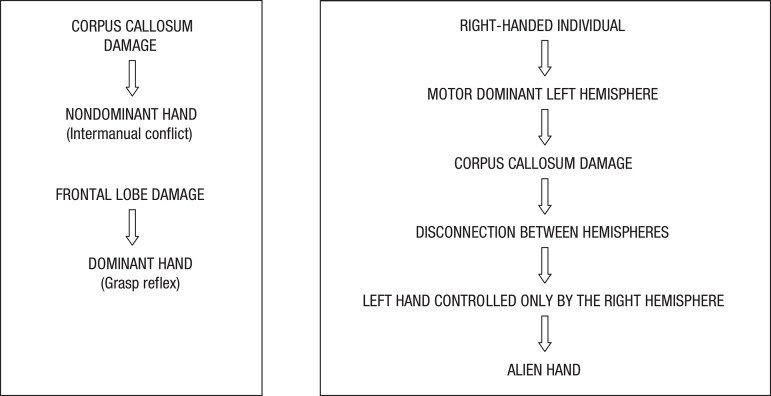
Proposed alien hand syndrome physiopathology.^4,15^

In the reviewed literature, all reported patients were right-handed or ambidextrous. Lesion and imaging studies have evidenced that, in right-handed individuals, the left hemisphere is dominant for complex or fine motor activities (reviewed in Geschwind et al., 1995).^15^ A disconnection between the left and right hemispheres in these individuals caused by damage to the corpus callosum, results in the left hand being controlled only by the right hemisphere (Figure 2), without governing by the motor dominant left hemisphere.^20^

Thus, this is a rare case report of a left-handed patient presenting alien hand syndrome which refutes the possibility proposed by Scepkowski and Cronin-Golomb^20^ that being left-handed, with the accompanying differences in brain organization relative to most right-handers, would preclude alien hand syndrome, but from an epidemiological standpoint may confirm that being left-handed could mitigate the development of alien-hand signs.

Toxoplasmosis is the most common etiology of cerebral mass lesion encountered in HIV-infected patients,^14^ being closely related to frontal lobe involvement.^21^ Hence, a higher association rate among AIDS, CNS toxoplasmosis and alien hand syndrome would be expected, where this was not observed in the reviewed literature, while no reports showing this association have been found.

Finally, we must consider that infectious treatable diseases offer an interesting model to study the evolution of alien hand syndrome as well as its neuroanatomical substrate because of the opportunity to monitor the correlation between disappearance of the syndrome and the associated structural modification during the resolution of the underlying pathological process. In our case for instance, alien hand sign disappeared with partial clinical and neuroimaging improvement, although a residual lesion remained in the fronto-parietal region.
